# Sugar signal mediates flavonoid biosynthesis in tea leaves

**DOI:** 10.1093/hr/uhac049

**Published:** 2022-03-14

**Authors:** Yi-Qing Lv, Da Li, Liang-Yu Wu, Yu-Meng Zhu, Ying Ye, Xin-Qiang Zheng, Jian-Liang Lu, Yue-Rong Liang, Qing-Sheng Li, Jian-Hui Ye

**Affiliations:** 1 Tea Research Institute, Zhejiang University, 866 Yuhangtang Road, Hangzhou 310058, China; 2 Institute of Sericulture and Tea, Zhejiang Academy of Agricultural Sciences, 298 Deshengzhong Road, Hangzhou 310021, China; 3 College of Horticulture, Fujian Agriculture and Forestry University, 15 Shangxiadian Road, Fuzhou 350000, China; 4 Ningbo Yinzhou District Agricultural Technical Extension Station, 55 Huifengxi Road, Ningbo 315100, China

## Abstract

Sugar metabolism and flavonoid biosynthesis vary with the development of tea leaves. In order to understand the regulatory mechanisms underlying the associations between them, a comprehensive transcriptomic analysis of naturally growing tea leaves at different stages of maturity was carried out. Based on weighted gene coexpression network analysis, the key gene modules (Modules 2 and 3) related to the varying relationship between sugar metabolism and flavonoid biosynthesis as well as the corresponding hub genes were obtained. KEGG (Kyoto Encyclopedia of Genes and Genomes) enrichment analysis showed that the transcription factors (TFs) in Modules 2 and 3 were mainly enriched in the pathway of plant hormone signal transduction. An *in vitro* study showed that the transcriptional levels of *ERF1B-like* TF for hexokinase inhibitor and sucrose treatments were upregulated, being respectively 28.1- and 30.2-fold higher than in the control, suggesting that *ERF1B-like* TFs participate in the sugar-induced regulation of flavonoid biosynthesis. The results of yeast one-hybrid and dual-luciferase assays demonstrated that *CsF3′H*, encoding flavonoid 3′-hydroxylase, was the target flavonoid biosynthetic gene for CsERF1B-like TF. Our study identified the potential key regulators participating in the metabolism of sugars and flavonoids, providing new insights into the crosstalk between sugar metabolism and flavonoid biosynthesis in tea plants.

## Introduction

Tea is a popular non-alcoholic beverage in the world; it is produced from the tender shoots of *Camellia sinensis* (L.) O. Kuntze, which contain plenty of secondary metabolites, e.g. polyphenols and alkaloids [1]. Flavonoids, as the main polyphenolic compounds in tea leaves, contribute to the bitterness and astringency of tea as well as health benefits like antioxidant, anticarcinogenic, and cardiovascular protective effects [2]. The abundant presence of flavonoids in young tea shoots is associated with multiple biological processes responding to abiotic stresses such as drought and light stress [3, 4].

The flavonoids present in fresh tea leaves mainly include two subclasses: flavanols and flavonols [1]. Catechins, including epigallocatechin gallate (EGCG), epicatechin (EC), epigallocatechin (EGC), epicatechin gallate (ECG), gallocatechin (GC), and catechin (C), are the predominant flavanol components in fresh tea leaves, while the majority of flavonols are the glycosyl derivatives of kaempferol, quercetin, and myricetin. The flavonoid composition of fresh leaves is affected by leaf maturity [5], cultivar [6], growing season [7], and cultivation conditions (e.g. shade) [3]. The biosynthesis of flavonoids is mediated by various signaling pathways, phytohormones, and transcription factors (TFs). There is increasing evidence for crosstalk among carbohydrates, phosphorus, phytohormones, light, and stress factor-induced signal transduction [8].

In plants, crosstalk between sucrose metabolism and flavonoid biosynthesis has been reported [9]. Sugars not only supply carbon skeletons as substrates for sink tissue growth, but also act as signal molecules or stimuli by influencing metabolic processes or regulating relevant gene expression [10]. Flavonoid biosynthesis comprises two biosynthetic pathways: phenylpropanoid and flavonoid biosynthesis pathways. Diverse flavonoid compounds have the same upstream enzymes, and downstream branches lead to the generation of flavanols, flavonols, and anthocyanins [5]. Sugar-induced accumulation of anthocyanins has been reported in many plants, e.g. grape berries [11], apple [12], and *Arabidopsis* [13]. Sugars enhance the transcription of many anthocyanin biosynthesis-related genes, such as *chalcone synthase* (*CHS*), *dihydroflavonol reductase* (*DFR*), and *UDP glucose-flavonoid-3-O-glycosyltransferases* (*UFGT*) [14–17]. Besides, phenylalanine ammonia lyase (PAL) activity was also stimulated by exogenous sucrose *in vitro* [18]. The glycosylation reaction catalyzed by UDP-glycosyltransferases is the ultimate step in flavonoid biosynthesis, altering the bioactivity, metabolism, and solubility of flavonoids in cells, whereby activated UDP-sugars from soluble sugars supply the sugar moiety of flavonol glycosides [5]. The impacts of exogenous sucrose treatment on the compositions of polyphenols and volatiles in tea plants have been investigated by transcriptomics and metabolomics [19]. Exogenous sugar treatment and *in vitro* culture of plant tissues are commonly used to artificially alter the sugar balance in plants for which transgenic lines are not readily available [11, 19]; however this may not represent the authentic natural growth of the plant, considering the sugar-induced osmotic stress occurring under *in vitro* treatment or that only specific forms of tissues or cells are used. The molecular mechanisms underlying sugar metabolism and diverse flavonoid biosynthesis in tea plants need elaboration.

Sucrose is the principal form of carbon efflux in plants, and hexokinase (HXK) is an important sugar sensor of signaling transduction [10]. To reveal the crosstalk between sucrose metabolism and flavonoid biosynthesis in naturally grown tea plants, we investigated the metabolism of soluble sugars and major flavonoids in spring tea shoots from transcriptional and metabolic aspects. Weighted gene coexpression network analysis (WGCNA) was used to investigate the associations between gene expression and the contents of soluble sugars and major flavonoids, and the hub genes in the key modules were obtained. Kyoto Encyclopedia of Genes and Genomes (KEGG) enrichment analysis was employed for achieving highly enriched TFs in the key modules of WGCNA. An *in vitro* study, using exogenous sucrose and hexokinase inhibitor, was employed to verify the regulatory effect of sucrose signal on the biosynthesis of flavonoids. The interactions between the candidate TFs and the promoters of structural genes in the flavonoid biosynthetic pathway were verified by yeast one-hybrid (Y1H) and dual-luciferase assays. Our study reveals the molecular mechanism underlying sugar signal-mediated flavonoid biosynthesis in tea leaves, providing a deep understanding of the crosstalk of flavonoid biosynthesis in tea plants.

## Results

### Compositions of soluble sugars and flavonoids varied with leaf maturity

**Figure 1:**
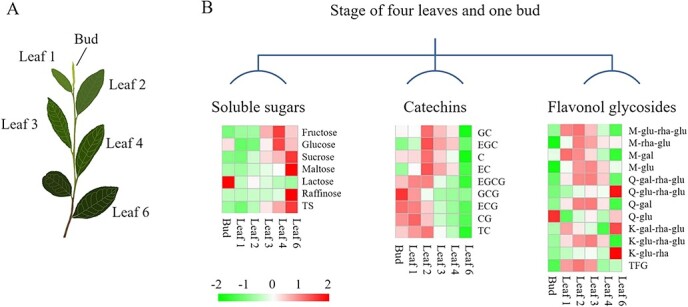
**a** Leaf positions and **b** visualization of differences in the pattern of soluble sugars, catechins, and flavonol glycosides in tea leaves at different stages of maturity

Figure 1a shows tea leaves at different stages of maturity, including Bud, Leaf 1, Leaf 2, Leaf 3, Leaf 4 and Leaf 6. The contents of soluble sugars in tea leaves are shown in Supplementary Data Table S1. Fructose, glucose, sucrose, maltose, lactose, and raffinose were detected in tea leaves, and sucrose accounted for 50–82% of total sugars (TS). This result agreed with previous work showing that sucrose was the major form of carbon efflux in tea plants [20]. The content of sucrose generally increased with leaf maturity, and the fully developed Leaf 6 had the highest sucrose content of 37.56 ± 3.12 mg/g dry weight (DW). Elevated photosynthetic activity of growing tea leaves resulted in the production of abundant sucrose for various physiological processes in tea plants. For flavonoids, Supplementary Data Table S2 shows that young leaves (Bud, Leaf 1 and Leaf 2) contained relatively high levels of total catechins (TC, 205.54 ± 17.52 to 259.74 ± 22.09 mg/g DW), while Leaf 6 contained the lowest level of TC (37.21 ± 8.11 mg/g DW), indicating that the TC level of tea leaves generally declined with leaf maturity. By contrast, the levels of total flavonol glycosides (TFG) were relatively stable, ranging from 2183 ± 317 to 3575 ± 484 μg/g DW for different tea leaves. Figure 1b shows the change patterns of soluble sugars, catechins, and flavonol glycosides in different leaf samples. At the stage of four leaves and a bud, the contents of sucrose and TS generally reached their highest levels in Leaf 4 and Leaf 6 compared with younger leaves, which was opposite to the change trends of catechins, indicating that flavonol glycosides exhibited discordant pattern as catechins.

### Global expression profiles are distinguished by leaf maturity

To provide systems-level insights into the correlations of sugar metabolism with flavonoid biosynthesis in tea leaves, Bud, Leaf 2, Leaf 4, and Leaf 6 were collected for transcriptome analysis. Supplementary Data Table S3 shows the statistical results of RNA sequencing and assembly. The mapping rates of reads to the reference genome (cv. ‘Shuchazao’) were >90% by TopHat. Supplementary Data Fig. S1A shows the high correlation coefficients (0.93–0.99) of the biological replicates of each sample, indicating a good quality of the replicates. Principal component analysis revealed a clear clustering of transcript profiles, corresponding to different leaf maturities. Leaf 2 and Leaf 4 samples were at a shorter distance, compared with that between Bud and Leaf 6, which was in line with the hierarchical clustering results. Supplementary Data Fig. S1D lists the numbers of differentially expressed genes (DEGs) between different samples compared in pairs. The pair of Leaf 6/Bud possessed the highest amount of DEGs, compared with the lowest amount of DEGs for the pair of Leaf 4/Leaf 2. The great number of DEGs observed in the pair of Leaf 6/Leaf 4 might be attributed to the fact that Leaf 6 was retained on the plant from last year. This also suggests that Leaf 2 and Leaf 4 had higher similarity in their transcriptional profiles compared with Bud and Leaf 6.

### Identification of key modules and genes related to sugar metabolism and flavonoid biosynthesis via coexpression analysis

**Figure 2:**
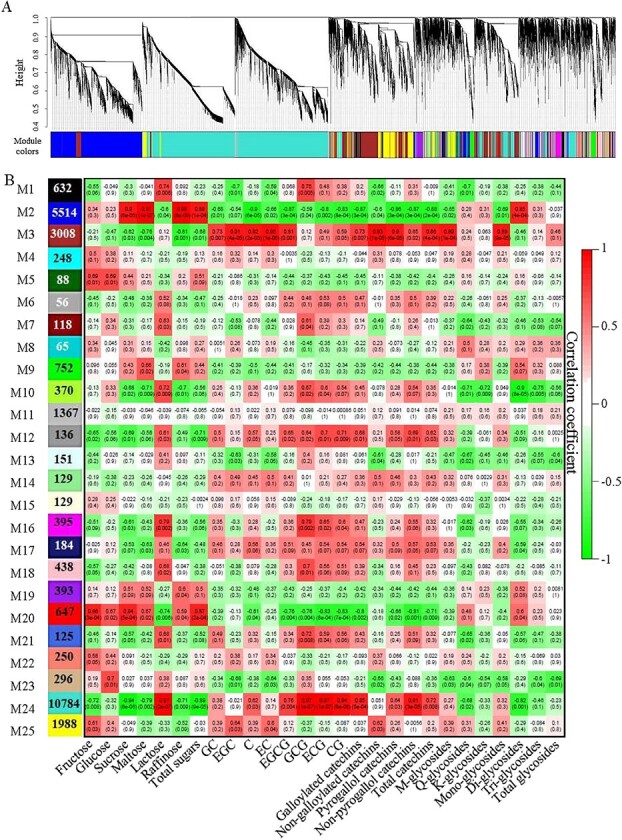
Correlations between chemical compositions and global gene expressions at different growth stages. **a** Hierarchical clustering dendrogram. **b** Modules labeled with different colors. Each cell includes the correlation coefficient (upper) and *P*-value (lower).

**Figure 3:**
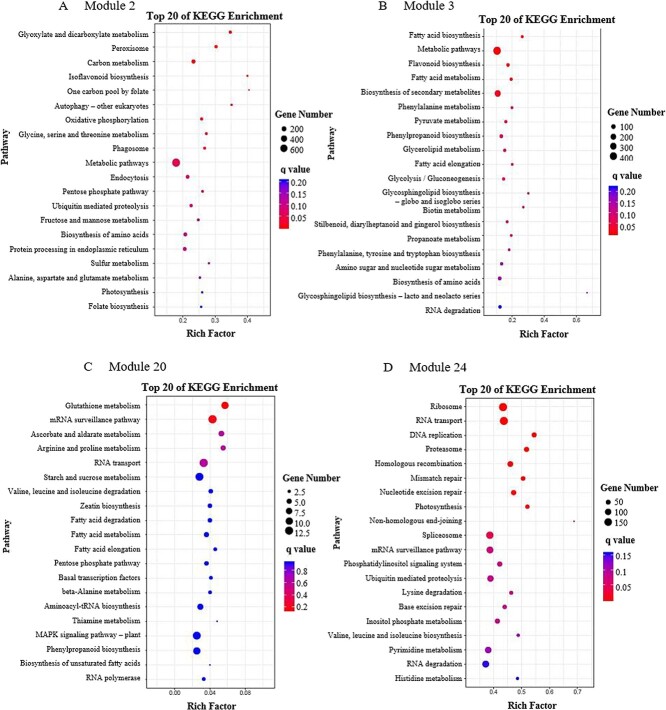
The top 20 KEGG-enriched pathways of genes in Module 2 (**a**), Module 3 (**b**), Module 20 (**c**), and Module 24 (**d**).

Network analysis provides a productive approach to visualizing and analyzing high-throughput biological data [21]. Coexpression analysis and network construction were carried out to obtain insights into the molecular mechanisms of the potential association between soluble sugars and flavonoids, and 25 individual modules (represented by various colors) are exhibited in the dendrogram in Fig. 2a. Eigengenes were applied to explore the relationships of modules and trait changes, the results of which are shown in Fig. 2b. Remarkably, the contents of sucrose, maltose, raffinose, and TS as well as diglycosides had positive correlations with the expressions of genes in Module 2 (blue, 5514 genes), with Pearson’s coefficients being >0.85, while the contents of catechin compounds (including different types) as well as M-glycosides and monoglycosides were negatively correlated with the gene expressions in Module 2 (blue). Similar change pattern was also observed in Module 20 (Red, 647 genes). By contrast, Module 3 (brown, 3008 genes) and Module 24 (turquoise, 10 784 genes) showed a general change pattern opposite to Module 2 and Module 20. KEGG enrichment analysis was performed to specify the significantly regulated pathways in Modules 2, 3, 20, and 24 (Fig. 3). The pathways of carbon metabolism and isoflavonoid biosynthesis were enriched in Module 2, while the flavonoid biosynthesis pathway was enriched in Module 3. By contrast, no sucrose-/flavonoid-related metabolic pathway was enriched in either Module 20 or Module 24. Hence, Modules 2 and 3 were selected for further analysis.

**Figure 4:**
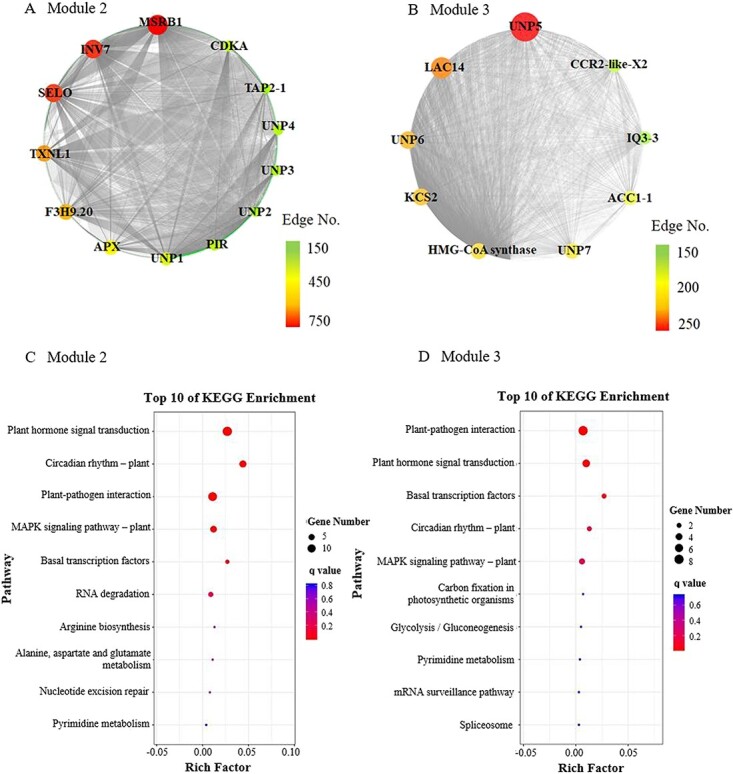
Network analysis for Module 2 (**a**) and Module 3 (**b**) and KEGG enrichment of the TFs in Module 2 (**c**) and Module 3 (**b**). UNP, unannotated protein; MSRB 1, peptide methionine sulfoxide reductase B1; SELO, selenoprotein O; TXNL 1, thioredoxin-like 1-1; APX, peroxisomal ascorbate peroxidase; INV 7, invertase 7; F3H9.20, F3H9.20 protein; PIR, protein PIR-like; TAP 2-1, peptide transporter 2 isoform 1; CDK A, cyclin dependent kinase A; KCS2, 3-ketoacyl-CoA synthase 2; HMG-CoA synthase, 3-hydroxy-3-methylglutaryl coenzyme A synthase; ACC 1-1, acetyl-CoA carboxylase 1 isoform 1; LAC14, laccase-14; CCR2-like-X2, cinnamoyl-CoA reductase 2-like isoform X2; IQ3-3, IQ-domain 3 isoform 3

The genes from Module 2 and Module 3 were used to construct the network. Thirteen candidate hub genes were obtained from Module 2 as the criteria of connectivity with >100 counts (Fig. 4a), including nine identified proteins and four unannotated proteins (UNPs), among which peptide methionine sulfoxide reductase B1 (MSRB1), selenoprotein O (SELO), thioredoxin-like 1-1 (TXNL1), and peroxisomal ascorbate peroxidase (APX) were involved in redox regulatory functions, while SELO and TXNL1 are located in chloroplasts [22–25]. Invertase 7 (INV7) was associated with carbohydrate metabolic process. Nine hub genes (six identified proteins and three UNPs) were obtained from Module 3 with the same criteria, among which 3-ketoacyl-CoA synthase 2 (KCS2), 3-hydroxy-3-methylglutaryl coenzyme A synthase (HMG-CoA synthase), and acetyl-CoA carboxylase 1 isoform 1 (ACC1-1), as well as laccase-14 (LAC14) and cinnamoyl-CoA reductase 2-like isoform X2 (CCR2-like-X2) were involved in the biosynthetic processes of fatty acids and phenolic compounds. Supplementary Data Table S4 lists the TF genes obtained from Modules 2 and 3, which were annotated based on the Tea Plant Information Archive (TPIA) database (http://tpdb.shengxin.ren/?from=timeline&isappinstalled=0). These TF genes were further analyzed by KEGG enrichment, and the pathway of plant hormone signal transduction was enriched in both Module 2 and Module 3 (Fig. 4c and d). There were seven TFs selected from Module 2 for further investigations based on the enriched pathway of plant hormone signal transduction, including [bZIP] TGA2.2 (TEA022601.1), [bZIP] TGA2.2 (TEA026302.1), [bZIP] UNP (TEA009167.1), [AP2/ERF-ERF] ERF 1B-like (TEA014156.1), PIF1-like (TEA006532.1), [bZIP] UNP (TEA030479.1), and bHLH144 (TEA001869.1).

### Validation of the regulatory effect of sugar signal on flavonoid biosynthesis

**Figure 5:**
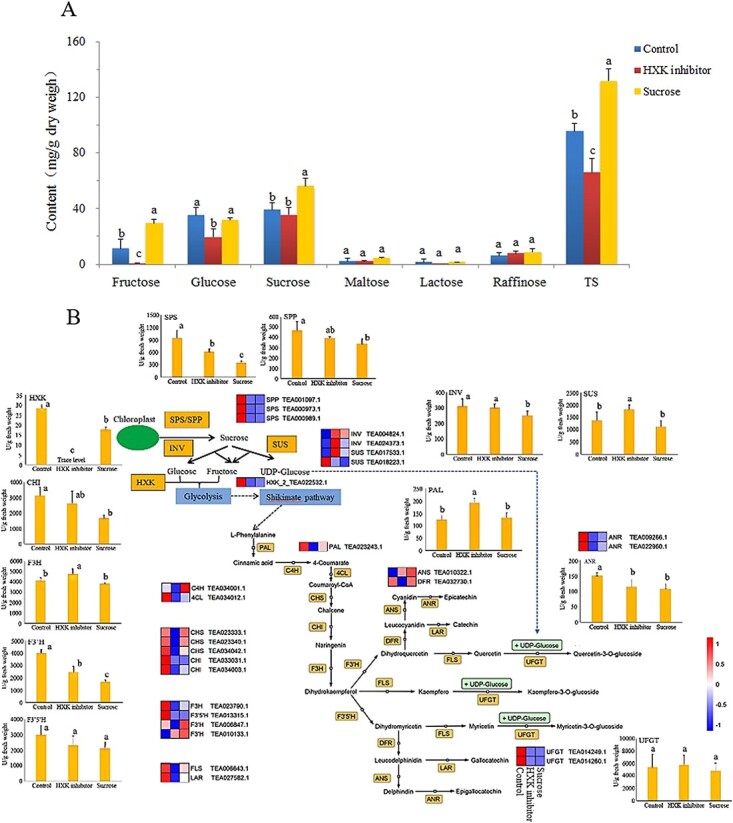
**a** Contents of soluble sugars in tea leaves under different treatments. **b** Visualization of transcriptional expression and activity of key enzymes in the pathway map of flavonoid biosynthesis associated with sucrose metabolism. The heat map was based on the value of log_2_ (fold change) from transcriptomic data sets. SPS, sucrose-phosphate synthase; SPP, sucrose-6-phosphate phosphatase; SUS, sucrose synthase; INV, invertase (β-fructofuranosidase); HXK, hexokinase; PAL, phenylalanine ammonia lyase; C4H, cinnamate 4-hydroxylase; 4CL, 4-coumaroyl CoA ligase; CHS, chalcone synthase; CHI, chalcone isomerase; F3H, flavanone 3-hydroxylase; F3′H, flavonoid 3′-hydroxylase; F3′5′H, flavonoid 3′,5′-hydroxylase; FLS, flavonol synthase; DFR, dihydroflavonol 4-reductase; LAR, leucoanthocyanidin reductase; ANR, anthocyanidin reductase; ANS, anthocyanidin synthase.

Sucrose is the major form of carbon efflux in the tea plant. To understand the impacts of sucrose metabolism on the biosynthesis of flavonoids, an *in vitro* study was carried out by using exogenous sucrose and HXK inhibitor to investigate the potential association between sucrose and flavonoid biosynthetic metabolisms. The compositions of soluble sugars and flavonoids in tea leaves under *in vitro* treatments are shown in Fig. 5a and Supplementary Data Table S5. The levels of glucose, sucrose, and TS in the control sample were much higher than those in the spring tea leaves (Supplementary Data Table S1), which might be attributed to the stronger photosynthetic activity of tea leaves during summer and autumn seasons. Under HXK inhibitor treatment, the content of TS was significantly lower than control, mainly due to the great decline of glucose and fructose (Fig. 5a). Thereby, HXK inhibitor may suppress the breakdown of sucrose into fructose and glucose. Under sucrose treatment, the tea leaves contained the highest amount of TS mainly due to the significant increases in sucrose and fructose. This suggests that exogenous sucrose could be transported to tea leaves under ambient sucrose stress and further metabolized into fructose and uridine diphosphate glucose (UDP-glucose) via sucrose synthase (SUS), which is also a plausible explanation for the stable glucose but largely increased fructose under exogenous sucrose treatment. Wu *et al.* [5] demonstrated that SUS-mediated breakdown is the ascendant cleavage way of imported sucrose in young tea leaves. Differential responses of sugar transporter genes to endogenous and exogenous sucrose were reported [26]. For flavonoid compounds, the HXK inhibitor-treated sample contained 127.72 ± 11.26 mg/g DW of TC, which was much less than control (176.54 ± 1.21 mg/g DW). This was attributed to the reduction of epi types of catechins (EGC, EC, EGCG, ECG), with maintaining rate ranging from 69.2 to 84.0% compared with control. Similarly, the content of TFG was also significantly decreased under HXK inhibitor treatment, mainly due to the decline of M-glycosides and Q-glycosides, which had maintaining rates of 82.9 and 72.9%, while K-glycosides were stable. These results suggest that inhibition of HXK and reduction of carbon flow into glycolysis were unfavorable for the biosynthesis of epi types of catechins as well as M-glycosides and Q-glycosides. However, the increases in fructose and sucrose in the sucrose-treated tea leaves did not consequentially lead to increases in TC and TFG. On the contrary, the content of TC was decreased upon sucrose treatment, mainly due to the significant reduction of EGCG (Supplementary Data Table S5). The contents of M-glycosides and K-glycosides slightly declined, whereas the content of Q-glycosides was increased 1.34-fold under exogenous sucrose treatment. With the same origin from dihydroquercetin as Q-glycosides, the contents of non-pyrogallol catechins, such as C, EC and CG, were also significantly elevated. Hence, excessive imported sucrose and fructose in tea leaves may not necessarily flow into the shikimate pathway and phenylpropanoid biosynthesis, followed by flavonoid biosynthesis pathway, but may potentially impact the biosynthesis of flavonoids through sugar signaling.

RNA sequencing and enzyme activity analyses were employed to reveal the underlying association between sugar metabolism and the biosynthesis of flavonoids. The results of RNA sequencing and assembly are shown in Supplementary Data Table S3 and the sample profiles are given in Supplementary Data Fig. S2. The principal component analysis result showed that different tea samples were well resolved, indicating substantial changes accrued in the tea leaves after *in vitro* treatments. The samples under HXK inhibitor and sucrose treatments had higher similarity in their transcriptional programs compared with control, which was in line with the hierarchical clustering results. The pair of sucrose/control possessed the highest number of DEGs, followed by the pair of HXK inhibitor/control, and the pair of HXK inhibitor/sucrose had the lowest number of DEGs (Supplementary Data Fig. S2B).

Figure 5b visualizes the transcriptional expressions and the activities of key enzymes in a pathway map of flavonoid biosynthesis associated with sucrose metabolism under different treatments. Both HXK inhibitor and sucrose treatments significantly suppressed the transcription of *HXK* genes, compared with control. The enzyme activity of HXK was completely inhibited by HXK inhibitor and partially suppressed by excessive sucrose in tea leaves. Hence, HXK inhibitor not only suppressed HXK activity, but also sufficiently inhibited the transcription of *HXK* gene. Excessive imported sucrose could also suppress the transcription of the *HXK* gene and adversely impact the activity of HXK enzyme. This explained the similarity of HXK inhibitor-treated and sucrose-treated samples based on the transcriptome. After glycolysis, the carbon flow entered into shikimate pathway, followed by phenylpropanoid biosynthesis initiated from l-phenylalanine. Furthermore, phenylpropanoids are channeled into flavonoid biosynthesis through the catalysis of upstream enzymes [CHS, chalcone isomerase (CHI), and flavanone 3-hydroxylase (F3H)]. Enzymes including CHI, F3H, DFR, anthocyanidin synthase (ANS), and anthocyanidin reductase (ANR) regulated the biosynthesis of catechins. Flavonol synthase (FLS) is committed to converting dihydroflavonols into flavonols, which are further conjugated with glucosyl groups via UFGT catalysis. The transcriptions of key flavonoid biosynthetic genes, such as *4-coumaroyl CoA ligase* (*4CL*), *CHI*, *F3H*, *flavonoid 3′,5′-hydroxylase* (*F3′5′H*), *FLS*, *LAR*, *ANR*, and *UFGT*, were mostly suppressed under HXK inhibitor and sucrose treatments, compared with control. However, the changes at transcriptional level may not consequentially lead to the changes in the corresponding enzyme activities. For example, the activities of UFGT were hardly affected by different treatments, while the transcriptional expressions of *UFGT* genes under HXK inhibitor and sucrose treatments were much lower than control. Besides, *PAL* was transcriptionally downregulated under HXK inhibitor treatment compared with control, while the activity of PAL was significantly higher than control. The transcriptional expressions of *cinnamate 4-hydroxylase* (*C4H*), *ANS*, and *flavonoid 3′-hydroxylase* (*F3′H*) were upregulated under sucrose treatment, compared with control and HXK inhibitor treatment. F3′H is the crucial enzyme channeled into the generation of non-pyrogallol catechins and Q-glycosides. The significantly elevated contents of EC, C, CG, and Q-glycosides were in agreement with the transcriptional change, although the activity of F3′H was even lower than those of control and under HXK inhibitor treatment. The flavonoid biosynthetic genes were more suppressed by HXK inhibitor treatment compared with control and sucrose treatment, which is consistent with the metabolic results showing that the HXK inhibitor-treated leaves had the lowest contents of TC and TFG (Supplementary Data Table S5). Regarding sucrose metabolism, the HXK inhibitor and sucrose treatments not only suppressed the transcription of *HXK*, but also affected the transcriptional expressions and activities of INV, SUS, and sucrose-phosphate synthase (SPS)/sucrose-6-phosphate phosphatase (SPP), possibly due to the feedback effect of hexoses. The elevated level of sucrose not only drove the breakdown of sucrose, but also adversely impacted the photosynthetic biosynthesis process of sucrose. Paul and Pellny [27] found that sugar accumulation in source leaves, due to source–sink imbalance, gave negative feedback on photosynthesis and plant productivity. Hexose accumulation in response to high carbon availability leads to the downregulation of photosynthetic gene expression [28].

### Validation of the interaction between the key transcription factor and flavonoid biosynthetic genes

**Figure 6:**
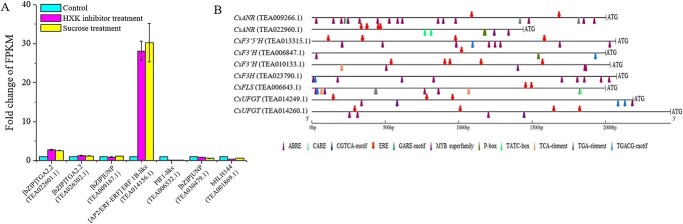
**a** Expression of selected transcription factors under different treatments. **b** The *cis*-acting elements in the promoters of key flavonoid biosynthetic genes. ERE, ethylene-responsive element; ABRE, ABA response element; TCA-element, salicylic acid responsive element; SARE, salicylic acid responsive element; CARE, 5′-CAACTC *cis*-element, TGA-element, auxin response element.

**Figure 7:**
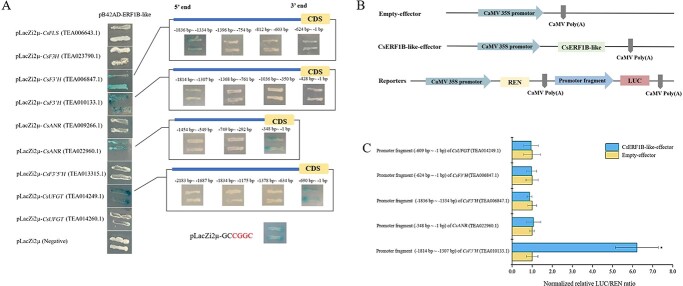
Characterization of CsERF1B-like interactions with the structural gene promoters of the flavonoid biosynthesis pathway in Y1H and dual-luciferase assays. **a** Interactions of CsERF1B-like proteins and the promoter fragments of *CsFLS*, *CsF3H*, *CsF3′Hs*, *CsANRs*, *CsF3′5′H*, and *CsUFGTs* in yeast cells. pLacZi2μ was used as a negative control. SD/Gal/Raf/−Ura−Trp/+X-gal medium indicates yeast nitrogen base containing galactose, raffinose, and X-gal, without Ura and Trp. **b** Construction diagrams of effector and reporter vectors for dual-luciferase assays. **c** Normalized relative LUC/REN ratio.

We investigated the effects of HXK inhibitor and exogenous sucrose on the transcriptional expressions of the seven candidate TFs selected above. Figure 6a shows that the expressions of *ERF1B-like* TF under HXK inhibitor and sucrose treatments were markedly upregulated 28.1- and 30.2-fold compared with control, without a significant difference between them. *TGA2.2* is associated with the salicylic acid- and auxin-inducible expressions of as-1-containing target promoters [29], the expression of which was also significantly upregulated by around 2.5-fold under HXK inhibitor and sucrose treatments. Thus, multiple hormone-induced signal transductions, especially ethylene-related signal transduction, participated in building the crosstalk between sugar metabolism and flavonoid biosynthesis. By contrast, the expressions of the *PIF1-like*, [*bZIP*] *UNP*, and *bHLH144* genes were significantly downregulated under HXK inhibitor and sucrose treatments, especially the *PIF1-like* gene, which was downregulated 0.09- and 0.11-fold, respectively. PIF1-like helicases were associated with the maintenance of genomic stability [30]. We cloned the promoters of the key flavonoid biosynthetic genes, including *CsFLS*, *CsF3′Hs*, *CsANRs*, *CsF3′5′H*, *CsF3H*, and *CsUFGTs*, and predicted the *cis*-elements as shown in Fig. 6b. The results show that ethylene-responsive elements (EREs) were universal in the promoter sequences of these genes. Furthermore, the Y1H assay was used to validate the interactions of the promoters of *CsFLS*, *CsF3′Hs*, *CsANRs*, *CsF3′5′H*, *CsF3H*, and *CsUFGTs* with CsERF1B-like TF. The finding that CsERF1B-like interacted with the promoters of *CsANR* (TEA022960.1), *CsF3′H* (TEA006847.1), *CsF3′H* (TEA010133.1), and *CsUFGT* (TEA014249.1) suggests that CsERF1B-like could regulate these genes by binding to their promoters (Fig. 7), while no interaction occurred between other structural genes and CsERF1B-like. To examine the CsERF1B-like-binding re gion of the promoters of these genes, the promoter regions of *CsANR* (TEA022960.1), *CsF3′H* (TEA006847.1), *CsF3′H* (TEA010133.1), and *CsUFGT* (TEA014249.1) were divided into three or four fragments, from the start codon to −2183 bp upstream, and each fragment was inserted into a pLacZi2μ vector. The results of the Y1H assay indicated that CsERF1B-like interacted with the fragments (−1836 to −1334 and −624 to −1 bp) of *CsF3′H* (TEA006847.1), the fragment (−1814 to −1307 bp) of *CsF3′H* (TEA010133.1), the fragment (−348 to −1 bp) of *CsANR* (TEA022960.1), and the fragment (−690 to −1 bp) of *CsUFGT* (TEA014249.1). Based on the prediction of the binding site of CsERF1B-like in the JASPAR database (https://jaspar.genereg.net/), we analyzed the coding sequences of all the fragments whether or not they interacted with CsERF1B-like, and found that the *cis*-acting element CGGC was present in all of the fragments that interacted with CsERF1B-like TF. The interaction of *cis*-acting element CGGC with CsERF1B-like was also demonstrated in yeast cells (Fig. 7a). A dual-luciferase assay was used to further validate the interactions between CsERF1B-like and these target promoter fragments. The effector and reporter vector construction diagrams are shown in Fig. 7b. Clearly, the overexpression of CsERF1B-like only dramatically increased the normalized relative ratio of firefly luciferase to *Renilla* luciferase (LUC/REN) of the promoter fragment (−1814 to −1307 bp) of *CsF3′H* (TEA010133.1) compared with control, suggesting that the transcription of *CsF3′H* (TEA010133.1) was activated by CsERF1B-like TF. This result was consistent with the higher transcriptional levels of *F3′H* (TEA010133.1) in the sucrose- and HXK inhibitor-treated samples compared with control (Fig. 5). No interaction occurred between other flavonoid biosynthetic genes (*CsUFGTs*, *CsANRs*, *CsFLS*, *CsF3′5′H*, and *CsF3H*) and CsERF1B-like TF based on the results of firefly luciferase complementation imaging and dual-luciferase assays (Supplementary Data Fig. S3A and B). Thus, our results indicated that CsERF1B-like TF upregulated the transcription of *CsF3′H* (TEA010133.1) in the pathway of flavonoid biosynthesis in tea leaves.

## Discussion

Sucrose, an important photosynthetic product of plants, supplies carbon skeletons or sugar moieties for glycosyl derivatives, and also acts as a signal molecule or stimulus to regulate various metabolic processes. Here, we depicted the transcriptomic and metabolic profiles of soluble sugars and flavonoids in naturally grown tea leaves at different stages of maturity, and obtained the gene modules closely correlated with the varying relationship between sugar metabolism and flavonoid biosynthesis via WGCNA. The candidate TFs were selected from the enriched pathway of plant hormone transduction according to the KEGG enrichment result of key Module 2. The regulatory effect of the sugar signal on the transcription of the candidate *ERF1B-like* TF was verified in the *in vitro* study using exogenous sucrose and HXK inhibitor. The results of Y1H and dual-luciferase assays demonstrated the binding of Cs ERF1B-like TF to the promoter of *CsF3′H* (TEA010133.1), which promoted the transcription of *CsF3′H* (TEA010133.1). Thus, the sugar signal induced the transcription of the hormone-related TF *CsERF1B-like* and further mediated the biosynthesis of flavonoids, implying that crosstalk is present among sugars, hormones, and flavonoids.

In addition to the distribution of sucrose between source leaves (e.g. developed leaves) and sink organs (e.g. young leaves) [31], HXK acts as a sugar sensor or is involved in intracellular sugar signal transduction to modulate the development of organs and growth [32, 33]. Accumulation of hexoses can be sensed by HXK [33]. In the *in vitro* study, we used exogenous sucrose and 2-acetamido-2-deoxy-d-glucose as an HXK inhibitor to alter the sugar metabolism balance in tea leaves. 2-Acetamido-2-deoxy-d-glucose is an N-acetylglucosamine and is a strong hexokinase inhibitor that does not specifically affect protein kinase activity [32]. Thus, although the sugar-sensing ability and the activity of HXK were diminished under HXK inhibitor treatment, the role in sugar signaling was hardly affected. The suppressed expression of *HXK* under both HXK inhibitor and sucrose treatments might play a similar role in the activation of sugar signaling transduction, which could be a plausible explanation for their higher similarity in the transcriptomic profiles compared with control, as well as the elevated transcriptional levels of *CsERF1B-like* TF in both samples. Thus, the promoted transcription of *CsERF1B-like* TF is more likely to be related to HXK-mediated sugar signaling transduction rather than the sugar-sensing ability of HXK, as well as the levels of sucrose and hexoses in tea leaves. Our study showed that no interaction occurred between CsERF1B-like TF and the promoters of *CsANR*, *CsUFGT*, *CsF3H*, *CsF3′5′H*, and *CsFLS*, but not *CsF3′H* (TEA010133.1). The binding of CsERF1B-like TF to the promoter fragment of *CsF3′H* (TEA010133.1) from −1814 bp to −1307 bp indicated that the transcription of *CsF3′H* could be induced by the sugar signal via CsERF1B-like TF. F3*′*H is the enzyme that converts dihydrokaempferol to dihydroquercetin, resulting in the generation of Q-glycosides and non-pyrogallol catechins. This was in line with the increases in Q-glycosides and non-pyrogallol catechins under sucrose treatment. However, the levels of Q-glycosides under HXK inhibitor treatment were greatly reduced, and the contents of TC and TFG were lower compared with sucrose-treated samples and control. This suggests that under carbon-deficient conditions, sucrose and its products as the material basis for the biosynthesis of flavonoids are more important for the accumulation of flavonoids compared with the effect of HXK-mediated sugar signal transduction. Therefore sucrose metabolism may exert a regulatory effect on the biosynthesis of flavonoids by providing a material basis and HXK-mediated sugar signal transduction involving with hormones. It was also reported that the transcription of *CsF3′5′H* in tea leaves was efficiently induced by sucrose [34].

Plant hormones participate in regulating flavonoid biosynthesis. Indole-3-acetic acid and 1-aminocyclopropane-1-carboxylic acid elevated the transcription of the flavonol pathway, promoting the accumulation of flavonols and modulating the levels of kaempferol and quercetin [35]. In red Chinese pear fruits, ethylene was reported to suppress the biosynthesis of anthocyanin [36]. Gibberellins, abscisic acid, and jasmonate modulated the sucrose-induced transcription of anthocyanin biosynthetic genes in *Arabidopsis* [37]. Application of exogenous brassinosteroid stimulated polyphenol biosynthesis in tea plants by increasing endogenous nitric oxide (NO) [38]. The hormone-induced accumulation of flavonoids may also involve carbon fixation and metabolism [39], and vice versa. Flavonoid compounds act as negative regulators of auxin transport [40], scavenging reactive oxygen species and providing defenses against herbivores and pathogens [41].

Our study linked ERF1B-like TF to sugar-induced regulation of flavonoid biosynthesis. As we know, the transcription of flavonoid biosynthetic genes is mediated by MYBs and MYB–bHLH–WDR complexes [42]. MYBs are important TFs regulating flavonoid biosynthesis, which were mainly present in the pathway of plant pathogen interaction in Module 3 in our study. Flavonoids contribute to the coloration of plant tissues or organs and enhance plant defenses [43]. The action of MdERF1B on MdMYB9 and MdMYB11 to mediate the accumulation of anthocyanin and proanthocyanidin was reported in apple (*Malus* × *domestica*) [44]. Wu *et al.* [45] identified *PbERF22* from ‘Zaosu’ pear, and testified that PbERF22 enhanced the activation effects of PbMYB10 and PbMYB10b on the promoter of *PbUFGT*. Our finding shows that CsERF1B-like can directly bind to the promoters of the key flavonoid biosynthetic gene *CsF3′H*. In citrus, it was reported that CitERF32 and CitERF33 directly bound to the promoter of *CitCHIL1* while CitRAV1 formed a transcription complex with CitERF33, strongly enhancing the accumulation of flavonoids [46]. Multiple hormone-mediated ethylene response factors(ERFs) are important agents whereby the sugar signal directly and indirectly modulates the biosynthesis of flavonoids, implying a crosstalk among flavonoid biosynthesis, sucrose metabolism, and phytohormones.

## Materials and methods

### Plant materials and sampling

Tea plants cv. ‘Longjing 43’ were cultivated in the tea garden of Zhejiang University, Hangzhou, China (30.27°N, 120.20°E). At the stage of four leaves and a bud (May 2019), the apical buds, second leaves, fourth leaves and sixth leaves (mature leaf from last year) basipetal from the apical bud were harvested, and the positions of the tea leaves were as indicated in Fig. 1a. Three biological replicates were used, and each biological replicate of tea leaves at the same position was collected from three to five plants. All the freshly plucked leaves were instantly immersed in liquid nitrogen for 30 minutes, and the frozen tea leaves were stored in a −80°C refrigerator prior to analyses.

For an *in vitro* study, tea cuttings with four leaves and a bud were obtained from cv. ‘Longjing 43’ in September 2019, and were placed in water for 24 hours. 2-Acetamido-2-deoxy-d-glucose (Baomanbio, Shanghai, China) was used as an HXK inhibitor for *in vitro* treatment according to previous studies [32]. The tea cuttings were equally divided into three treatment groups: (i) control: placing in water for 20 hours; (ii) HXK inhibitor treatment: placing in 5% (w/v) HXK inhibitor solution for 20 hours; and (iii) exogenous sucrose treatment: placing in 5% (w/v) sucrose solution for 20 hours, based on a previous study [47]. The third leaves of each group were collected for chemical, transcriptomic, and enzymatic activity analyses. Three biological replicates were obtained from each of four tea cuttings. The fresh leaves were treated with liquid nitrogen as above.

### Analysis of major flavonoids in tea leaves

The frozen leaves were lyophilized and then ground into powder. One hundred and fifty milligrams of the obtained tea powder was extracted with 25 ml of 50% ethanol solution (70°C, 20 minutes). After centrifugation (4°C, 12 000 rpm, 10 minutes), the supernatant was submitted to liquid chromatographic analyses of catechins and flavonol glycosides [5]. Catechins were quantified using external standards, while flavonol glycosides were quantified using the corresponding aglycones.

### Determination of soluble sugars

Five hundred milligrams of the obtained tea powder was extracted using 6 ml of 50% ethanol solution (v/v) for 30 minutes at room temperature, with a 1-minute ultrasonic treatment every 4 minutes. The mixture was submitted to centrifugation (4°C, 4500 rpm, 15 minutes), and the supernatant was collected and stored at 4°C. Prior to UHPLC–MS/MS analysis, the samples were centrifuged (4°C, 12 000 rpm, 10 minutes), and the supernatant was collected. The UHPLC conditions were: Acquity UPLC BEH Amide column (2.1 mm × 150 mm, 1.7 μm); column temperature of 35°C; injection volume of 3 μl; mobile phase A = 0.1% ammonium hydroxide + 99.9% water (v/v); mobile phase B = 0.1% ammonium hydroxide + 99.9% acetonitrile (v/v); gradient elution program, from 18% A/82% B (v/v) to 35% A/65% B (v/v) for the first 7.6 minutes, to 28% A/72% B (v/v) at 8 minutes, to 30% A/70% B (v/v) at 9 minutes, maintained at 30% A/70% B (v/v) for another 6 minutes (from 9 to 15 minutes), followed by 3 minutes re-equilibrium; flow rate of 0.15 ml/minute. An electron spray ionization (ESI) technique was employed for MS scan in negative ion mode. The ion source conditions were the same as in our previous work [1], except cone voltage was set to 25 V. Single-ion monitoring (SIM) mode was used for quantifying fructose ([M-H]^−^ 179 *m*/*z*) and glucose ([M-H]^−^ 179 *m*/*z*), sucrose ([M-H]^−^ 341 *m*/*z*), lactose ([M-H]^−^ 341 *m/z*), maltose ([M-H]^−^ 341 *m*/*z*), and raffinose ([M-H]^−^ 503 *m*/*z*), using an external standard method.

### Protein extraction and enzyme activity measurement

The total protein was extracted from tea leaves with the M5 Plant Protein Extraction Kit (Mei5 Biotechnology, Co., Ltd, Beijing, China). The activities of enzymes, including SPP, SPS, neutral INV, SUS, CHI, F3H, ANR, F3′H, UFGT, and F3′5′H, were measured using the corresponding ELISA kits (Shanghai SUER Biological Technology, Co., Ltd, Shanghai, China). The activity of HXK was measured with a hexokinase colorimetric assay kit (Sigma–Aldrich, St Louis, MO, USA).

### Transcriptomic analysis

RNA isolation and sequencing were carried out by Personal Biotechnology Co., Ltd (Shanghai, China). The cDNA libraries were obtained and checked for quality, and an Illumina HiSeq™ 2500 platform was used for PCR amplification and sequencing. Moreover, the obtained clean reads were mapped to the reference genome sequence of *C. sinensis* cv. ‘Shuchazao’ [48] using HISAT2. 2.4, and then were assembled by StringTie. The value of fragments per kilobase per million (FPKM) was used for quantification and normalization of gene abundance. The differential expressions of mRNAs were analyzed using DESeq2 software. The DEGs were identified using fold change >2 and false discovery rate (FDR) <0.05 as cutoff values (*P* < 0.05).

### Quantitative real-time PCR analysis

First-strand cDNA was synthesized for each of the RNA samples (1 μg). Specific primers (Supplementary Data Table S6) were designed by NCBI Primer-BLAST based on the genome sequences of *C. sinensis* cv. ‘Shuchazao’. Quantitative real-time PCR (qPCR) cycling was run on an Applied Biosystems™ StepOnePlus™ Real-Time PCR System (Applied Biosystems™ ABI, Carlsbad, CA, USA) as follows: 95°C for 2 minutes; 40 cycles at 95°C for 3 seconds; 60°C for 30 seconds. β-Actin was used as an internal control. Technical replicates were performed in triplicate. Supplementary Data Fig. S4 shows high correlations between the qPCR results and the corresponding transcriptomic data (*R*^2^ = 0.836 for naturally growing tea leaves, *R*^2^ = 0.890 for the *in vitro* study), indicating that the transcriptomic dataset can represent the transcript abundances.

### Coexpression network and bioinformatic analyses

The WGCNA package was used for constructing the gene coexpression network, by using the available R implementation (soft-thresholding power β = 10). Modules in different colors were obtained using the DynamicTreeCut algorithm. Cytoscape software (version 3.8.0) was used to visualize the coexpression networks by using selected genes. The KEGG database was applied to enrich the metabolic pathways of selected modules.

### Extraction of genomic DNA, cloning of promoter, and identification of *cis*-acting elements

*F3H* (TEA023790.1), *ANR* (TEA009266.1, TEA022960.1), *F3′5′H* (TEA013315.1), *F3′H* (TEA006847.1, TEA010133.1), *FLS* (TEA006643.1), and *UFGT* (TEA014249.1, TEA014260.1) gene sequences were retrieved from the TPIA, and the primers are shown in Supplementary Data Table S6. Genomic DNA of tea leaf samples was extracted. The fragment of the promoter (~2000 bp) was amplified from the cv. ‘Longjing 43’ genome DNA by PCR. The PCR program was: 98°C for 3 minutes; 98°C for 10 seconds, 60°C for 15 seconds; 72°C for 2 minutes, 35 cycles, and 72°C for 7 minutes. The purified PCR product was ligated into a pLacZiμ vector and transformed into *Escherichia coli* DH5α competent cells for sequencing. The *cis*-elements located in the cloned promoter were predicted by the PlantCARE online site.

### Y1H assay

The promoter fragments of the target genes were cloned and constructed into the pLacZi2μ vector. The coding sequence of *Cs ERF1B-like* was inserted into the pB42AD vector [49]. The primers used to construct vectors are listed in Supplementary Data Table S6. The Y1H assay was conducted according to the method previously described [50]. Briefly, the constructed pLacZi2μ vector and pB42AD vector were transformed into yeast strain EGY48, and empty vector was transformed as the negative control. SD/Glu/−Ura−Trp minimal medium containing 0.11 M glucose (Glu), without uracil (Ura) or tryptophan (Trp), was used for growing the yeast cells. After culture for 72 hours, positive clones were selected and further cultured in selective SD/Gal/Raf/−Ura−Trp/+X-gal medium plates containing 0.11 M galactose (Gal), 0.02 M raffinose (Raf), 10× buffered salt (0.26 M Na_2_HPO_4_•7H_2_O, 0.25 M NaH_2_PO_4_), 0.1 mM X-gal, and without Ura or Trp, for stringent screening of possible interactions between ERF1B-like and the promoters characterized by blue products.

### Firefly luciferase complementation imaging and dual-luciferase assays

The promoters of the above flavonoid biosynthetic genes were constructed into the pGreenII 0800-LUC vector and transformed into GV3101 (pSoup-p19, *Agrobacterium tumefaciens*) as reporters. The coding sequence of containing pGreenII 62-SK vector was transformed into GV3101(pSoup-p19) as an effector, while the empty pGreenII 62-SK vector transformed into GV3101(pSoup-p19) was the control. Different groups were infiltrated into leaves of tobacco (*Nicotiana benthamiana*). After 3 days (long-day white light illumination), the infiltrated tobacco leaves were collected. For imaging, 0.5 mM luciferin (Shanghai Macklin Biochemical Co., Ltd, China) was injected into the infiltrated place and the leaves were kept for 6 minutes in dark. LUC imaging was performed on the Lumazone PyLoN20488 system. The Dual Luciferase Reporter Gene Assay Kit, purchased from Yeasen Biotechnology (Shanghai) Co., Ltd, China, was used for analysis of the relative LUC/REN ratio to show the interactions between CsERF1B-like and the promoters. Each leaf was measured for each construct pair, using three leaves for a biological replicate. The relative LUC/REN ratio of the experimental group was normalized based on the control group.

### Statistical analysis

All the tests were carried out in triplicate, and the results were presented as mean value ± standard deviation. Analysis of significant differences was performed using SPSS Statistics 22 software (IBM Inc., Chicago, IL, USA), using the Tukey test. The heat map was plotted using heatmap2 of the R package.

## Acknowledgements

We thank Dr. Jin-Xi Huo for the kind offer of pB42AD and pLacZi2μ vectors and technical support in the yeast one-hybrid assay. This study was supported by the National Natural Science Foundation of China (Project No. 31600554), the Major Project of Agricultural Science and Technology in Breeding of Tea Plant Variety in Zhejiang Province (Project No. 2021C02067), and the Discipline Startup Fund of Zhejiang Academy of Agricultural Sciences (10405080121CC2201G).

## Author contributions

Y.Q.L. and D.L. performed the experiments and wrote the manuscript, with guidance from J.H.Y., X.Q.Z, J.L.L. and Y.R.L. L.Y.W., Y.Y. and Y.M.Z. performed the chemical analysis work. Q.S.L. and J.H.Y. designed the study, supervised the project, and revised the manuscript.

## Data availability

The data that support the findings of this study are openly available within this manuscript and its supporting materials. The RNA-sequencing (CRA002901 for naturally growing tea shoots) and RNA-sequencing (CRA002902 for *in vitro* study) raw data were uploaded to the BIG data center (https://bigd.big.ac.cn/) with the project No. PRJCA002956.

## Conflict of interest

The authors declare that they have no conflict of interest.

## Supplementary data

Supplementary data is available at *Horticulture Research* online.

## Supplementary Material

Web_Material_uhac049

## References

[1] Zheng XQ , Nie Y, Gao Y et al. Screening the cultivar and processing factors based on the flavonoid profiles of dry teas using principal component analysis. J Food Compos Anal. 2018;67:29–37.

[2] Wang HF , Provan CJ, Helliwell K. Tea flavonoids: their functions, utilisation and analysis. Trends Food Sci Technol. 2000;11:152–60.

[3] Wang YS , Gao LP, Shan Y et al. Influence of shade on flavonoid biosynthesis in tea [*Camellia sinensis* (L.) O. Kuntze]. Sci Hortic. 2012;141:7–16.

[4] Wang WD , Xin HH, Wang ML et al. Transcriptomic analysis reveals the molecular mechanisms of drought-stress-induced decreases in *Camellia sinensis* leaf quality. Front Plant Sci. 2016;7:385. 27066035 10.3389/fpls.2016.00385 PMC4811933

[5] Wu LY , Fang ZT, Lin JK et al. Complementary iTRAQ proteomic and transcriptomic analyses of leaves in tea plant (*Camellia sinensis* L.) with different maturity and regulatory network of flavonoid biosynthesis. J Proteome Res. 2019;18:252–64. 30427694 10.1021/acs.jproteome.8b00578

[6] Shen JZ , Zou ZW, Zhang XZ et al. Metabolic analyses reveal different mechanisms of leaf color change in two purple-leaf tea plant (*Camellia sinensis* L.) cultivars. Hortic Res. 2018;5:7. 29423237 10.1038/s41438-017-0010-1 PMC5802758

[7] Liu M , Tian HL, Wu JH et al. Relationship between gene expression and the accumulation of catechin during spring and autumn in tea plants (*Camellia sinensis* L.). Hortic Res. 2015;2:15011. 26504566 10.1038/hortres.2015.11 PMC4595990

[8] Leon P , Sheen J. Sugar and hormone connections. Trends Plant Sci. 2003;8:110–6. 12663220 10.1016/S1360-1385(03)00011-6

[9] Morkunas I , Narozna D, Nowak W et al. Cross-talk interactions of sucrose and *Fusarium oxysporum* in the phenylpropanoid pathway and the accumulation and localization of flavonoids in embryo axes of yellow lupine. J Plant Physiol. 2011;168:424–33. 21056513 10.1016/j.jplph.2010.08.017

[10] Smeekens S , Hellmann HA. Sugar sensing and signaling in plants. Front Plant Sci. 2014;5:113. 24723932 10.3389/fpls.2014.00113 PMC3972449

[11] Dai ZW , Meddar M, Renaud C et al. Long-term *in vitro* culture of grape berries and its application to assess the effects of sugar supply on anthocyanin accumulation. J Exp Bot. 2014;65:4665–77. 24477640 10.1093/jxb/ert489 PMC4115254

[12] Liu XJ , An XH, Liu X et al. MdSnRK1.1 interacts with MdJAZ18 to regulate sucrose-induced anthocyanin and proanthocyanidin accumulation in apple. J Exp Bot. 2017;68:2977–90. 28549152 10.1093/jxb/erx150 PMC5853841

[13] Jeong SW , Das PK, Jeoung SC et al. Ethylene suppression of sugar-induced anthocyanin pigmentation in *Arabidopsis*. Plant Physiol. 2010;154:1514–31. 20876338 10.1104/pp.110.161869 PMC2971625

[14] Tsukaya H , Ohshima T, Naito S et al. Sugar-dependent expression of the *CHS-A* gene for chalcone synthase from *Petunia* in transgenic *Arabidopsis*. Plant Physiol. 1991;97:1414–21. 16668565 10.1104/pp.97.4.1414 PMC1081180

[15] Gollop R , Even S, Colova-Tsolova V et al. Expression of the grape dihydroflavonol reductase gene and analysis of its promoter region. J Exp Bot. 2002;53:1397–409. 12021287

[16] Solfanelli C , Poggi A, Loreti E et al. Sucrose-specific induction of the anthocyanin biosynthetic pathway in *Arabidopsis*. *Plant Physiol* 2006;140:637–46. 10.1104/pp.105.072579 PMC1361330 16384906

[17] Das PK , Shin DH, Choi SB et al. Cytokinins enhance sugar-induced anthocyanin biosynthesis in *Arabidopsis*. Mol Cells. 2012;34:93–101. 22699753 10.1007/s10059-012-0114-2 PMC3887782

[18] Morkunas I , Marczak L, Stachowiak J et al. Sucrose-induced lupine defense against *Fusarium oxysporum* sucrose-stimulated accumulation of isoflavonoids as a defense response of lupine to *Fusarium oxysporum*. Plant Physiol Biochem. 2005;43:363–73. 15907688 10.1016/j.plaphy.2005.02.011

[19] Qian YM , Zhang SX, Yao SB et al. Effects of *vitro* sucrose on quality components of tea plants (*Camellia sinensis*) based on transcriptomic and metabolic analysis. BMC Plant Biol. 2018;18:121. 29914362 10.1186/s12870-018-1335-0 PMC6007066

[20] Yue C , Cao HL, Wang L et al. Effects of cold acclimation on sugar metabolism and sugar-related gene expression in tea plant during the winter season. Plant Mol Biol. 2015;88:591–608. 26216393 10.1007/s11103-015-0345-7

[21] Iancu OD , Kawane S, Bottomly D et al. Utilizing RNA-Seq data for *de novo* coexpression network inference. Bioinformatics. 2012;28:1592–7. 22556371 10.1093/bioinformatics/bts245 PMC3493127

[22] Mullen RT , Lisenbee CS, Miernyk JA, Trelease RN. Peroxisomal membrane ascorbate peroxidase is sorted to a membranous network that resembles a subdomain of the endoplasmic reticulum. Plant Cell. 1999;11:2167–85. 10559442 10.1105/tpc.11.11.2167 PMC144122

[23] Weissbach H , Etienne F, Hoshi T et al. Peptide methionine sulfoxide reductase: structure, mechanism of action, and biological function. Arch Biochem Biophys. 2002;397:172–8. 11795868 10.1006/abbi.2001.2664

[24] Dangoor I , Peled-Zehavi H, Levitan A et al. A small family of chloroplast atypical thioredoxins. Plant Physiol. 2009;149:1240–50. 19109414 10.1104/pp.108.128314 PMC2649386

[25] Fichman Y , Koncz Z, Reznik N et al. SELENOPROTEIN O is a chloroplast protein involved in ROS scavenging and its absence increases dehydration tolerance in *Arabidopsis thaliana*. Plant Sci. 2018;270:278–91. 29576081 10.1016/j.plantsci.2018.02.023

[26] Wei XY , Nguyen STT, Collings DA et al. Sucrose regulates wall ingrowth deposition in phloem parenchyma transfer cells in *Arabidopsis* via affecting phloem loading activity. J Exp Bot. 2020;71:4690–702. 32433727 10.1093/jxb/eraa246

[27] Paul MJ , Pellny TK. Carbon metabolite feedback regulation of leaf photosynthesis and development. J Exp Bot. 2003;54:539–47. 12508065 10.1093/jxb/erg052

[28] Wang L , Dong Q, Zhu QD et al. Conformational characteristics of rice hexokinase OsHXK7 as a moonlighting protein involved in sugar signalling and metabolism. Protein J. 2017;36:249–56. 28555318 10.1007/s10930-017-9718-x

[29] Niggeweg R , Thurow C, Kegler C, Gatz C. Tobacco transcription factor TGA2.2 is the main component of as-1-binding factor ASF-1 and is involved in salicylic acid- and auxin-inducible expression of as-1-containing target promoters. J Biol Chem. 2000;275:19897–905. 10751419 10.1074/jbc.M909267199

[30] Boule JB , Zakian VA. Roles of Pif1-like helicases in the maintenance of genomic stability. Nucleic Acids Res. 2006;34:4147–53. 16935874 10.1093/nar/gkl561 PMC1616966

[31] Ho LH , Klemens PAW, Neuhaus HE et al. SlSWEET1a is involved in glucose import to young leaves in tomato plants. *J Exp Bot* 2019;70:3241–54. 10.1093/jxb/erz154 PMC6598072 30958535

[32] Hofmann M , Roitsch T. The hexokinase inhibitor glucosamine exerts a concentration dependent dual effect on protein kinase activity *in vitro*. J Plant Physiol. 2000;157:13–6.

[33] Moore B , Zhou L, Rolland F et al. Role of the *Arabidopsis* glucose sensor HXK1 in nutrient, light, and hormonal signaling. Science. 2003;300:332–6. 12690200 10.1126/science.1080585

[34] Wang YS , Xu YJ, Gao LP et al. Functional analysis of flavonoid 3′,5′-hydroxylase from tea plant (*Camellia sinensis*): critical role in the accumulation of catechins. BMC Plant Biol. 2014;14:347. 25490984 10.1186/s12870-014-0347-7 PMC4275960

[35] Lewis DR , Ramirez MV, Miller ND et al. Auxin and ethylene induce flavonol accumulation through distinct transcriptional networks. Plant Physiol. 2011;156:144–64. 21427279 10.1104/pp.111.172502 PMC3091047

[36] Ni JB , Zhao Y, Tao RY et al. Ethylene mediates the branching of the jasmonate-induced flavonoid biosynthesis pathway by suppressing anthocyanin biosynthesis in red Chinese pear fruits. Plant Biotechnol J. 2020;18:1223–40. 31675761 10.1111/pbi.13287 PMC7152598

[37] Loreti E , Povero G, Novi G et al. Gibberellins, jasmonate and abscisic acid modulate the sucrose-induced expression of anthocyanin biosynthetic genes in *Arabidopsis*. New Phytol. 2008;179:1004–16. 18537890 10.1111/j.1469-8137.2008.02511.x

[38] Li X , Zhang L, Ahammed GJ et al. Nitric oxide mediates brassinosteroid-induced flavonoid biosynthesis in *Camellia sinensis* L. J Plant Physiol. 2017;214:145–51. 28482335 10.1016/j.jplph.2017.04.005

[39] Li X , Zhang L, Ahammed GJ et al. Salicylic acid acts upstream of nitric oxide in elevated carbon dioxide-induced flavonoid biosynthesis in tea plant (*Camellia sinensis* L.). Environ Exp Bot. 2019;161:367–74.

[40] Brown DE , Rashotte AM, Murphy AS et al. Flavonoids act as negative regulators of auxin transport *in vivo* in *Arabidopsis*. Plant Physiol. 2001;126:524–35. 11402184 10.1104/pp.126.2.524 PMC111146

[41] Peer WA , Murphy AS. Flavonoids and auxin transport: modulators or regulators? *Trends Plant Sci* 2007;12:556–63. 10.1016/j.tplants.2007.10.003 18198522

[42] Xu WJ , Dubos C, Lepiniec L. Transcriptional control of flavonoid biosynthesis by MYB-bHLH-WDR complexes. *Trends Plant Sci* 2015;20:176–85. 10.1016/j.tplants.2014.12.001 25577424

[43] Lu YF , Chen Q, Bu YF et al. Flavonoid accumulation plays an important role in the rust resistance of *Malus* plant leaves. Front Plant Sci. 2017;8:1286. 28769974 10.3389/fpls.2017.01286 PMC5514348

[44] Zhang J , Xu HF, Wang N et al. The ethylene response factor MdERF1B regulates anthocyanin and proanthocyanidin biosynthesis in apple. Plant Mol Biol. 2018;98:205–18. 30182194 10.1007/s11103-018-0770-5

[45] Wu T , Liu HT, Zhao GP et al. Jasmonate and ethylene-regulated ethylene response factor 22 promotes lanolin-induced anthocyanin biosynthesis in 'Zaosu' pear (*Pyrus bretschneideri* Rehd.) fruit. Biomolecules. 2020;10:278. 10.3390/biom10020278 PMC7072184 32054132

[46] Zhao CN , Liu XJ, Gong Q et al. Three AP2/ERF family members modulate flavonoid synthesis by regulating type IV chalcone isomerase in citrus. Plant Biotechnol J. 2021;19:671–88. 33089636 10.1111/pbi.13494 PMC8051604

[47] Zhang SH , Peng FT, Xiao YS et al. Peach PpSnRK1 participates in sucrose-mediated root growth through auxin signaling. Front Plant Sci. 2020;11:409. 32391030 10.3389/fpls.2020.00409 PMC7193671

[48] Wei CL , Yang H, Wang SB et al. Draft genome sequence of *Camellia sinensis* var. *sinensis* provides insights into the evolution of the tea genome and tea quality. Proc Natl Acad Sci USA. 2018;115:E4151–8. 29678829 10.1073/pnas.1719622115 PMC5939082

[49] Lin RC , Ding L, Casola C et al. Transposase-derived transcription factors regulate light signaling in *Arabidopsis*. Science. 2007;318:1302–5. 18033885 10.1126/science.1146281 PMC2151751

[50] Zhang H , Zhang Q, Zhai H et al. IbBBX24 promotes the jasmonic acid pathway and enhances fusarium wilt resistance in sweet potato. Plant Cell. 2020;32:1102–23. 32034034 10.1105/tpc.19.00641 PMC7145486

