# Supplementary material for: Sugar signal mediates flavonoid biosynthesis in tea leaves
Source: Hortic Res. 2022 Mar 14;9:uhac049. doi: 10.1093/hr/uhac049 (PMC9113228; doi:10.1093/hr/uhac049)
Supplement: Web_Material_uhac049 [file web_material_uhac049.zip › 6Supplementary materials.pdf]

Table S1 The contents of soluble sugars in naturally growing tea leaves with different maturity (mg/g dry weight)

Compounds	Bud	Leaf 1	Leaf 2	Leaf 3	Leaf 4	Leaf 6
Fructose	0.80±0.25^{c}	1.08±0.21^{c}	1.13±0.14^{c}	2.52±0.04^{b}	3.50±0.35^{a}	2.31±0.56^{b}
Glucose	4.48±0.82^{b}	0.35±0.06^{c}	0.73±0.10^{c}	4.13±0.31^{b}	8.09±0.39^{a}	5.26±0.84^{b}
Sucrose	6.58±0.53^{d}	5.45±0.67^{d}	13.92±0.12^{c}	21.45±1.01^{b}	24.86±2.53^{b}	37.56±3.12^{a}
Maltose	0.38±0.04^{c}	0.27±0.01^{c}	0.73±0.07^{bc}	0.99±0.06^{bc}	1.22±0.10^{b}	3.83±0.71^{a}
Lactose	0.81±0.06^{a}	0.09±0.02^{cd}	0.24±0.04^{b}	0.15±0.01^{c}	0.07±0.01^{cd}	0.01±0.01^{d}
Raffinose	0.07±0.06^{b}	0.01±0.01^{b}	0.24±0.05^{b}	0.25±0.02^{b}	0.55±0.13^{b}	4.79±0.72^{a}
TS	13.11±0.91^{de}	7.24±0.43^{e}	16.99±0.19^{d}	29.48±1.11^{c}	38.29±3.45^{b}	53.76±5.66^{a}

TS: total sugars. Data with different alphabetic letters in the same column were significantly different at $P<0.05$.

Table S2 The contents of flavonoids in tea leaves with different maturity

Compounds	Bud	Leaf 1	Leaf 2	Leaf 3	Leaf 4	Leaf 6
Catechins (mg/g dry weight)						
GC	2.71±0.99[a]	2.67±1.02[a]	4.01±0.35[a]	3.25±0.17[a]	2.87±0.19[a]	0.52±0.07[b]
EGC	11.46±2.83[cd]	19.58±4.17[c]	48.75±4.95[a]	38.26±4.28[ab]	33.06±4.48[b]	7.5±1.82[d]
C	0.92±0.22[ab]	0.94±0.30[ab]	1.28±0.17[a]	0.96±0.08[ab]	0.78±0.07[b]	0.08±0.02[c]
EC	5.16±0.08[d]	8.18±0.92[c]	13.33±1.25[a]	10.76±1.05[b]	8.99±1.12[bc]	2.45±0.48[e]
EGCG	125.75±9.66[a]	150.93±12.76[a]	151.17±11.71[a]	92.83±9.44[b]	69.82±8.84[b]	20.14±4.44[c]
GCG	5.43±0.06[a]	3.71±0.29[b]	1.83±0.24[c]	0.65±0.07[d]	0.40±0.01[d]	0.26±0.06[d]
ECG	52.29±3.60[a]	49.56±3.38[a]	37.84±3.25[b]	23.05±2.20[c]	16.36±1.99[c]	6.08±1.19[d]
CG	1.83±0.08[ab]	2.09±0.19[a]	1.52±0.15[b]	0.91±0.10[c]	0.62±0.09[c]	0.18±0.04[d]
TC	205.54±17.52[bc]	237.66±23.03[ab]	259.74±22.09[a]	170.67±17.37[cd]	132.89±16.78[d]	37.21±8.11[e]
Total flavonol glycosides (μg/g dry weight)						
M-glu-rha-glu	34±6[bc]	89±14[a]	94±13[a]	75±4[a]	48±8[b]	17±3[c]
M-rha-glu	14±1[b]	33±4[a]	45±6[a]	42±2[a]	36±6[a]	35±7[a]
M-gal	452±60[b]	735±101[a]	685±91[a]	549±30[ab]	382±69[b]	149±4[c]
M-glu	56±8[b]	144±19[a]	186±25[a]	192±11[a]	162±29[a]	140±27[a]
Q-gal-rha-glu	108±17[b]	224±30[a]	293±39[a]	298±16[a]	246±43[a]	109±20[b]
Q-glu-rha-glu	38±5[c]	86±12[bc]	138±19[b]	167±9[b]	148±26[b]	372±63[a]
Q-gal	83±16[b]	144±20[a]	193±26[a]	196±11[a]	146±27[a]	49±9[b]
Q-glu	69±10[a]	28±4[c]	42±6[bc]	47±3[b]	38±5[bc]	52±9[ab]
K-gal-rha-glu	1087±166[bc]	1439±195[ab]	1285±169[abc]	1180±62[abc]	858±148[c]	1628±292[a]
K-glu-rha-glu	222±31[c]	447±60[b]	575±86[ab]	620±32[a]	467±81[ab]	39±7[d]
K-glu-rha	20±3[c]	30±5[bc]	39±5[bc]	42±2[b]	32±6[bc]	99±17[a]
TFG	2183±317[b]	3399±463[a]	3575±484[a]	3408±183[a]	2562±446[ab]	2688±451[ab]

GC: gallocatechin; EGC: epicatechin gallate; C:catechin; EC: epicatechin; EGCG: epigallocatechin gallate; GCG: gallocatechin gallate; ECG: epicatechin gallate; CG: catechin gallate; TC: total catechins; M-gal-rha-glu: myricetin galactosyl-rhamnosyl-glucoside; M-gal: myricetin galactoside; M-glu: myricetin glucoside; K-glu-rha-gal: kaempferol glucosyl-rhamnosyl-galactoside; K-gal: kaempferol galactoside; K-glu-rha: kaempferol glucosyl-rhamnoside; K-glu: kaempferol glucoside; Q-gal-rha-glu: quercetin galactosyl-rhamosyl-glucosied; Q-glu-rha-rha: quercetin glucosyl-rhamnosyl-rhamnoside; Q-glu-rha-glu: quercetin glucosyl-rhamnosyl-glucoside; Q-gal: quercetin galactoside; Q-glu: quercetin glucoside; TFG: total flavonol glycosides. Flavonol glycosides were quantified by the corresponding aglycones. Data with different alphabetic letters in the same column were significantly different at $P<0.05$.

Table S3 The statistics of RNA-sequencing and assembly results

Sample	Raw data (bp)	Clean data (bp)	Raw reads (Number)	Clean reads (Number)	Reads matched to reference genome
Natural growth					
Bud-1	6602351784	6092885938	43724184	40350238	36882440 (91.41%)
Bud-2	6756888204	6249774938	44747604	41389238	37821821 (91.38%)
Bud-3	6862031014	6353095782	45443914	42073482	38510987 (91.53%)
Leaf 2-1	6606846148	6125827796	43753948	40568396	37183570 (91.66%)
Leaf 2-2	7936788916	7365585814	52561516	48778714	44771311 (91.78%)
Leaf 2-3	7086216184	6571730494	46928584	43521394	39880071 (91.63%)
Leaf 4-1	6468173184	5994184788	42835584	39696588	35944170 (90.55%)
Leaf 4-2	6629281124	6148920226	43902524	40721326	37308961 (91.62%)
Leaf 4-2	6878829764	6371264404	45555164	42193804	38800375 (91.96%)
Leaf 6-1	6032158872	5599482566	39948072	37082666	33823032 (91.21%)
Leaf 6-2	6800596664	6333009762	45037064	41940462	38572971 (91.97%)
Leaf 6-3	6864707036	6378990470	45461636	42244970	38734167 (91.69%)
In vitro study					
Control-1	6913129800	6270642900	46087532	41804286	37855156 (90.55%)
Control-2	6730078200	6077243700	44867188	40514958	36610568 (90.36%)
Control-3	6949735800	6267568800	46331572	41783792	37846183 (90.58%)
Inhibitor-1	5957624100	5377308300	39717494	35848722	32452119 (90.53%)
Inhibitor-2	6389323200	5791675500	42595488	38611170	34979690 (90.59%)
Inhibitor-3	6742164300	6076763700	44947762	40511758	36706775 (90.61%)
Sucrose-1	5935552500	5345574900	39570350	35637166	32102549 (90.08%)
Sucrose-2	6205275900	5588392500	41368506	37255950	33677577 (90.40%)
Sucrose-3	5906299200	5329212600	39375328	35528084	32095914 (90.34%)

Inhibitor: HXK inhibitor.

Table S5 The contents of flavonoids in Leaf3 under exogenous sucrose and HXK inhibitor treatments

	Control	HXK inhibitor	Sucrose
Catechins (mg/g dry weight)			
GC	3.67±0.08[a] (100%)	3.05±0.33[b] (83.1%)	3.79±0.02[a] (103.3%)
EGC	40.49±0.24[a] (100%)	28.00±2.61[b] (69.2%)	37.80±2.07[a] (93.4%)
C	0.71±0.02[c] (100%)	0.92±0.02[b] (129.6%)	1.18±0.05[a] (166.2%)
EC	11.53±0.03[b] (100%)	9.68±0.68[c] (84.0%)	12.46±0.35[a] (108.1%)
EGCG	98.34±0.84[a] (100%)	69.10±6.50[b] (70.3%)	74.40±3.07[b] (75.7%)
GCG	0.61±0.01[c] (100%)	0.75±0.01[b] (123.0%)	0.80±0.02[a] (131.1)
ECG	20.64±0.17[a] (100%)	15.41±1.07[b] (74.7%)	20.46±0.71[a] (99.1%)
CG	0.55±0.02[b] (100%)	0.81±0.06[a] (147.3%)	0.78±0.01[a] (141.8%)
TC	176.54±1.21[a] (100%)	127.72±11.26[c] (72.3%)	151.69±6.12[b] (85.9%)
Flavonol glycosides (μg/g dry weight)			
M-glu-rha-glu	51±1[a] (100%)	49±2[a] (96.1%)	42±2[b] (82.4%)
M-rha-glu	17±5[b] (100%)	19±4[b] (111.8%)	28±2[a] (164.7%)
M-gal	404±8[a] (100%)	332±4[c] (82.2%)	377±20[b] (93.3%)
M-glu	180±4[a] (100%)	152±4[b] (84.4%)	176±8[a] (97.8%)
Q-gal-rha-glu	259±5[a] (100%)	176±6[b] (68.0%)	273±10[a] (105.4%)
Q-glu-rha-glu	131±1[b] (100%)	96±4[c] (73.3%)	195±8[a] (148.9%)
Q-gal	68±2[b] (100%)	61±2[b] (89.7%)	132±6[a] (194.1%)
Q-glu	29±2[b] (100%)	22±1[c] (75.9%)	55±2[a] (189.7%)
K-gal-rha-glu	307±20[a] (100%)	314±18[a] (102.3%)	292±34[a] (95.1%)
K-glu-rha-glu	96±1[a] (100%)	95±8[a] (99.0%)	72±11[b] (75.0%)
K-glu-rha	ND	ND	ND
TFG	1541±24[a] (100%)	1315±19[b] (85.3%)	1643±100[a] (106.6%)

GC: gallocatechin; EGC: epicatechin gallate; C: catechin; EC: epicatechin; EGCG: epigallocatechin gallate; GCG: gallocatechin gallate; ECG: epicatechin gallate; CG: catechin gallate; TC: total catechins; M-gal-rha-glu: myricetin galactosyl-rhamnosyl-glucoside; M-gal: myricetin galactoside; M-glu: myricetin glucoside; K-glu-rha-gal: kaempferol glucosyl-rhamnosyl-galactoside; K-gal: kaempferol galactoside; K-glu-rha: kaempferol glucosyl-rhamnoside; K-glu: kaempferol glucoside; Q-gal-rha-glu: quercetin galactosyl-rhamosyl-glucosied; Q-glu-rha-rha: quercetin glucosyl-rhamnosyl-rhamnoside; Q-glu-rha-glu: quercetin glucosyl-rhamnosyl-glucoside; Q-gal: quercetin galactoside; Q-glu: quercetin glucoside; TFG: total flavonol glycosides. Flavonol glycosides were quantified by the corresponding aglycones. Data with different alphabetic letters in the same column were significantly different at $P<0.05$.

Table S6 The primers sequence for qPCR and promoter cloning

Type	Gene name	Gene ID		primer (5’→3’)
qPCR	β-actin	TEA019484.1	F	CTTCCTCATGCTATCCTCCGTCTT
			R	ATTTCCCGTTCAGCAGTGGTG
	INV	TEA004824.1	F	GGACCCAAGCTGGACTAACC
			R	AGCCCTCTGAAGCCAAAACC
	SUS	TEA017533.1	F	ATCCCACGCAGAATGACGAG
			R	TAGCACTCTACCAGCCCTGT
	HXK1	TEA005890.1	F	CAATGCAGCGTATGTGGAGC
			R	AGATGCGATGAGCGGAAGTT
	HXK2	TEA022532.1	F	CCACATCTGGAAGGACGATTAT
			R	CTCAGCATCCATGTCCCTATC
	PAL	TEA023243.1	F	ACGACAACCCCTTGATCGAC
			R	TTGATGCCAAAGCCAGCCTA
	C4H	TEA034001.1	F	TCAAGGACACGAGGTTGCAG
			R	TGGGTGGTTGACGAGTTCTG
	4CL	TEA034012.1	F	TTCATGCGGAACTGTGGTCA
			R	TGGAGCCAACCATCCACATC
	CHS	TEA023340.1	F	GCAGGACATGGTTGTGGTTG
			R	TTGACTGATGGGCGAAGACC
	CHI	TEA033031.1	F	AGTTGAATGCGGTTGGGTTG
			R	CGCACCAGCATTCCTACATC
	F3H	TEA023790.1	F	ACTCAAGATGGCCCGACAAG
			R	CCTTCTCAAGGCCCATAGCC
	F3’H	TEA006847.1	F	CACCCATCAACCCCACTCTC
			R	GCCACCAGGTAGGAATCGTT
	DFR	TEA032730.1	F	TGCCAGTTGTGTCGTTCTC
			R	AGCAAACCCTTCTCTCTGC
	ANS	TEA010322.1	F	AACAAGCGAGTACGCAAAGC
			R	TGAAGCTCTTCCATGCCTCC
	ANR	TEA022960.1	F	TATGCGGTCAACACCACTGT
			R	TGTTCATCGGTGAGGTCTGC
	LAR	TEA027582.1	F	GCCTACGTACCTTCTCGTCC
			R	CAACGTCATGCCCAAACTCC
	FLS	TEA006643.1	F	CCCTCGGAGTTGAACCTCAC
			R	ACGACAAACACAGCCCAAGA
	UFGT	TEA014249.1	F	CGTATTCCACGCCACCTGTA
			R	AGCTCGGCTTCTCTGAACTG
	F3’5’H	TEA013315.1	F	GATTGCGTGGATGGACTTGC
			R	ATCCATCTCGTCGTGTGCTC
	ERF1B-like	TEA014156.1	F	GTTCACCTGTGGCGACTTTA
			R	CAACTCCACCATATTCCCTCTC

Promoter cloning	FLS	TEA006643.1	F	CACATAGCTCTTTTGTTGTG
			R	TGCACTCTCTCTACCTCCAT
	F3H	TEA023790.1	F	ATACACCGACCGTAACACGA
			R	AGCGTTGTTGTTGGCGCCAT
	F3’H-1	TEA006847.1	F	CATGAGACAAATCGGAGACT
			R	GCGATCGAGTGGTGAGGCAT
	F3’H-2	TEA010133.1	F	GGTGATGGAATGAGGAATGAGG
			R	TAGTTGTAGGTGGTGTACTT
	ANR-1	TEA009266.1	F	GACCAGACATTGCATATGCAG
			R	TGTTGTTGTTGTTGCCATTG
	ANR-2	TEA022960.1	F	GGATGATTGCCTCGGATTAC
			R	CTCAAGCAACAACTTGATGA
	F3’5’H	TEA013315.1	F	GTCTTCACGATTGGGCCACC
			R	AAGACTGTGTCTAGGGCCAT
	UFGT-1	TEA014249.1	F	TTGGTAACACACACGAGAGG
			R	GCTTCTCTGAACTGTTCCAT
	UFGT-2	TEA014260.1	F	CGTGCACTCCCACGAGGACA
			R	GTGATGCTGACTCCATATTA
	ERF1B-like	TEA014156.1	F	ATGGATTCCTCTGTATTCCACTCTC
			R	TCAGCAAGGACTAGTACTCTGACTTGA

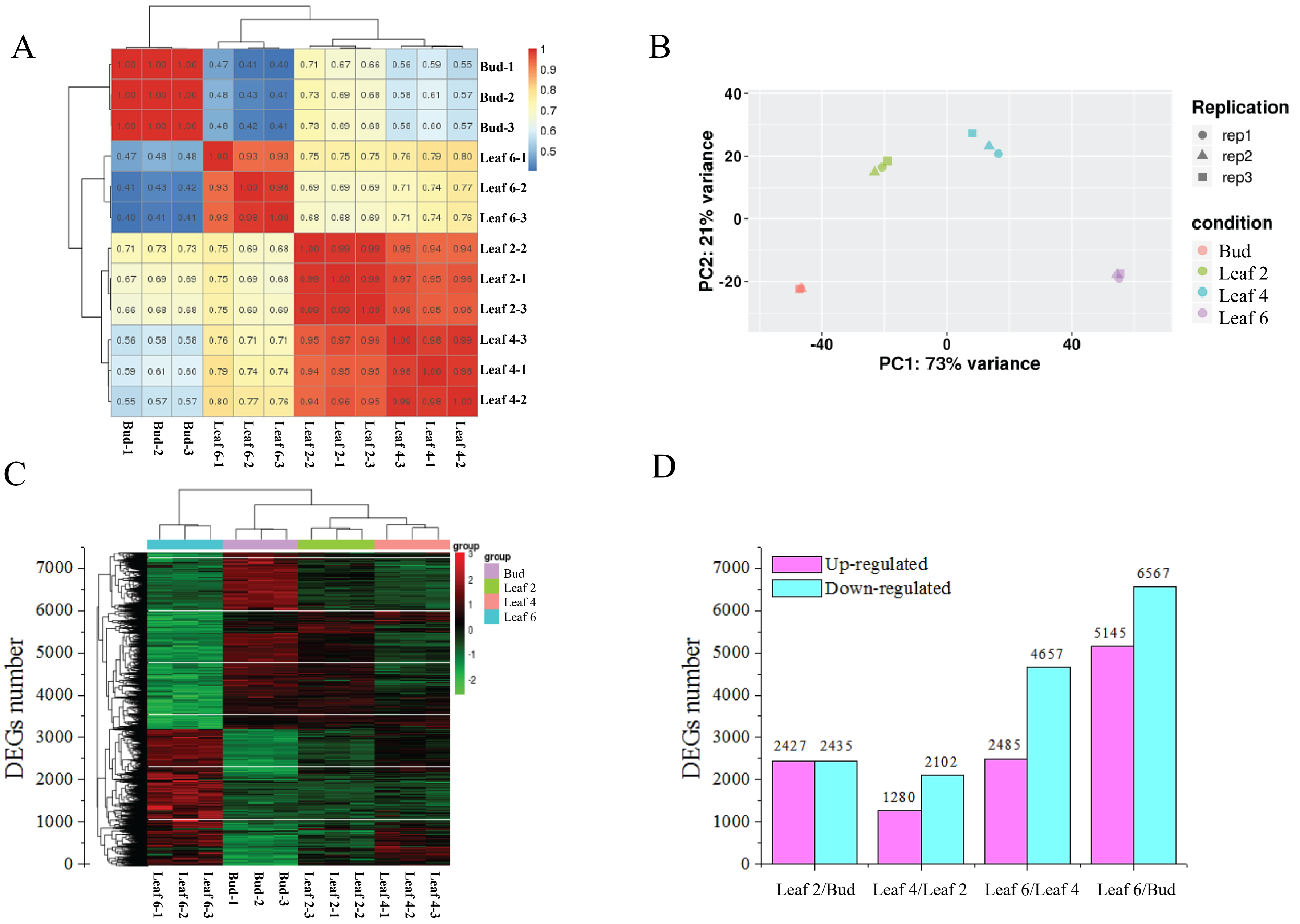

Fig. S1 Correlation analysis (A) and principal component analysis (B) of three replicates of different samples. Hierarchical clustering (C) and DEGs numbers of the fresh tea leaves with different maturity (D).

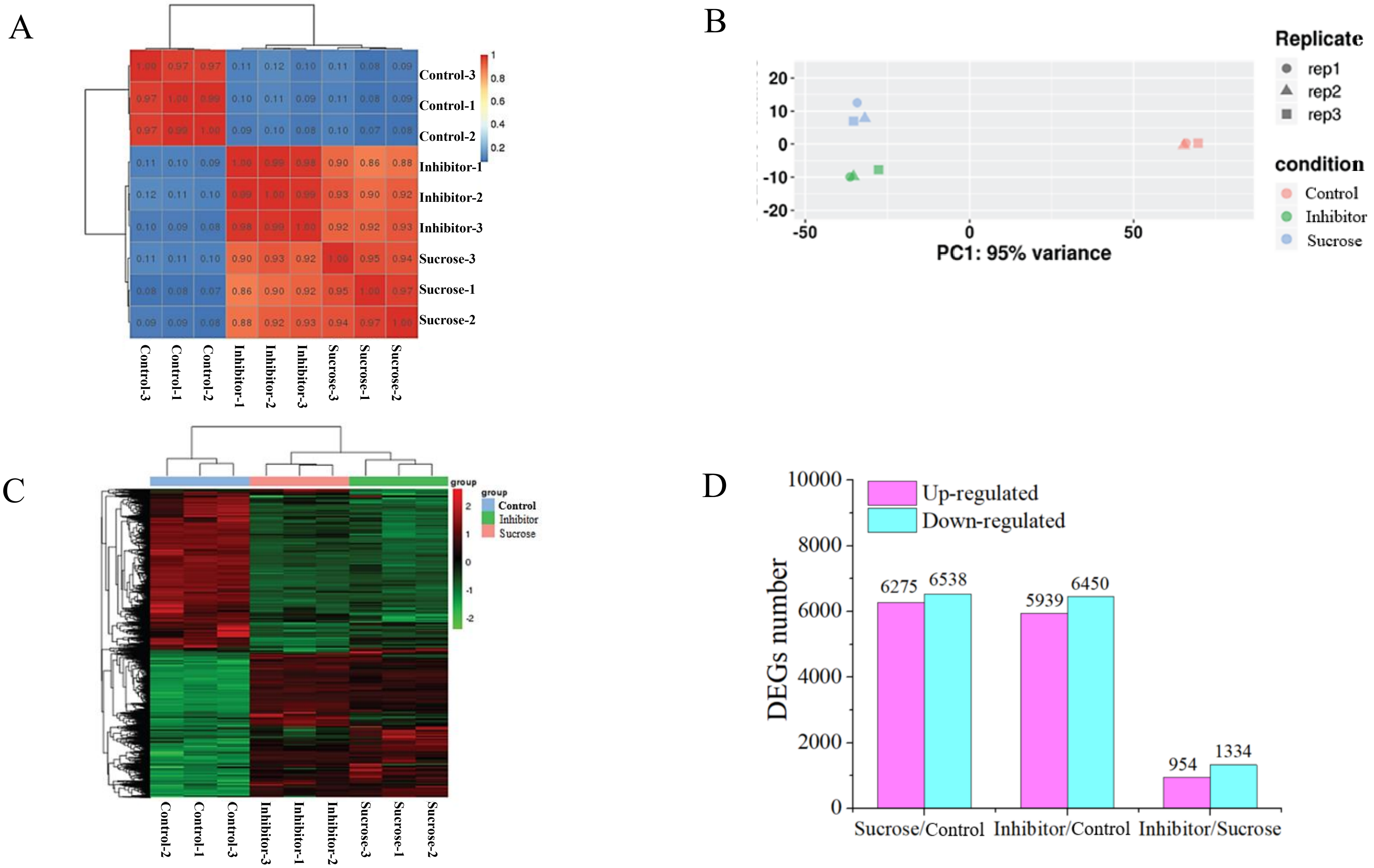

Fig. S2 Correlation analysis (A) and principal component analysis (B) of three replicates of different samples. Hierarchical clustering (C) and DEGs numbers of the fresh tea leaves under different treatments (D).

A

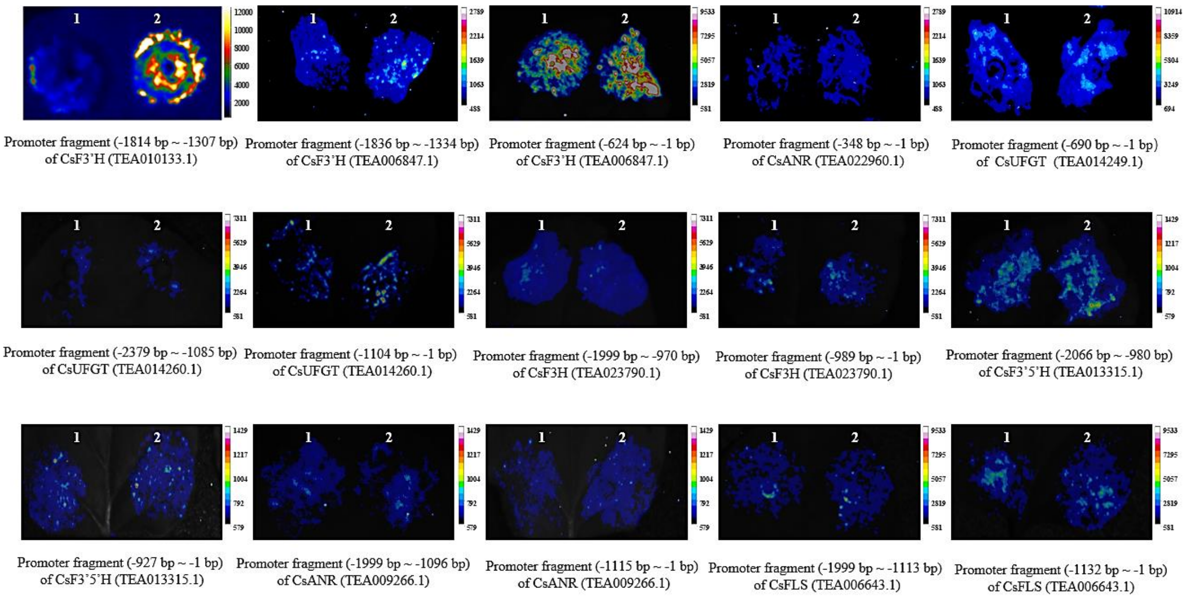

B

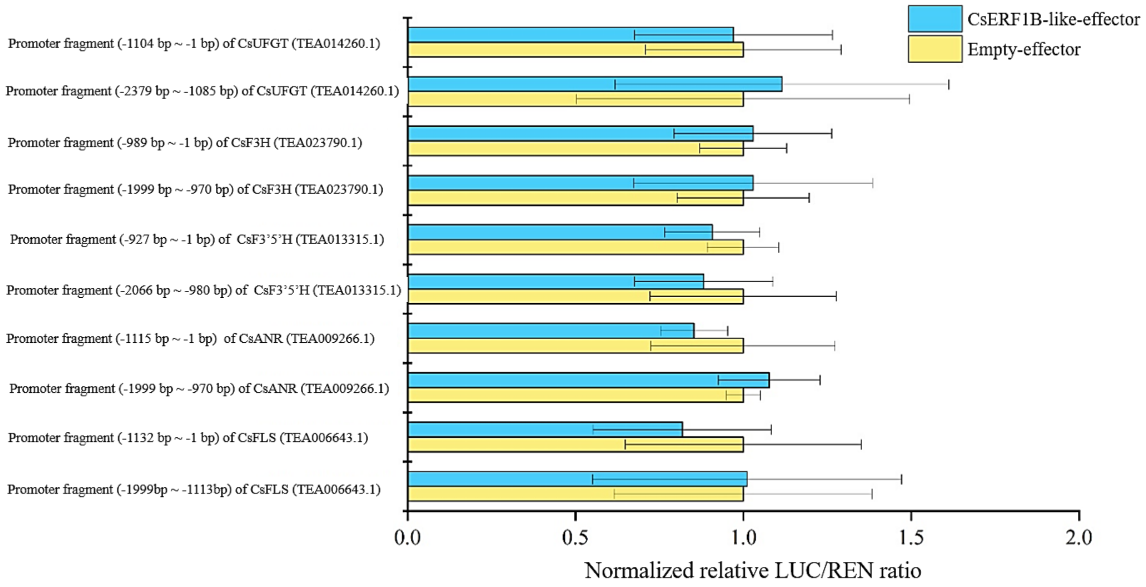

Fig. S3 Characterization of CsERF1B-like interactions with the promoters of structural genes in the flavonoids biosynthetic pathway based on Firefly luciferase complementation imaging (LCI) assay (A) and Dual-luciferase assay (B). 1: Control; 2: CsERF1B-like+promoter fragment of structural gene in the flavonoid biosynthetic pathway

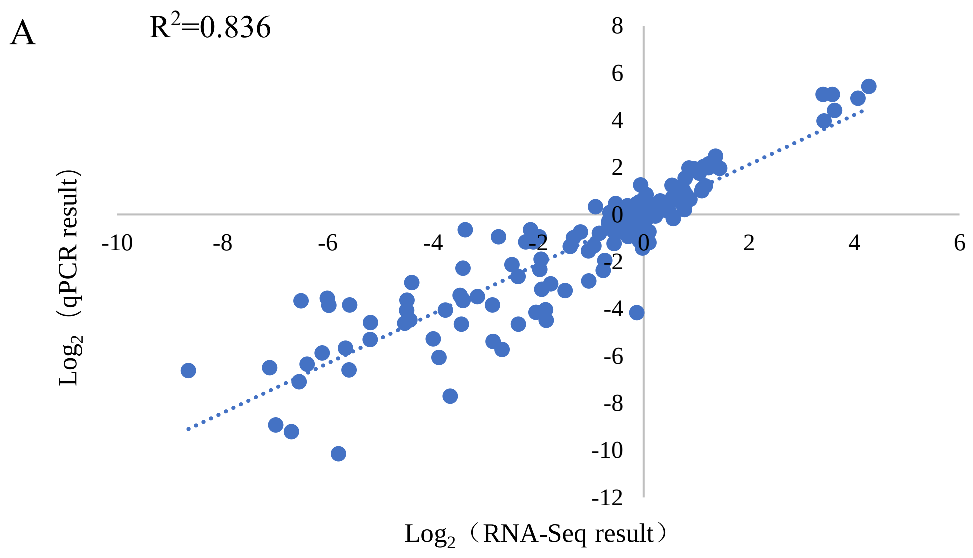

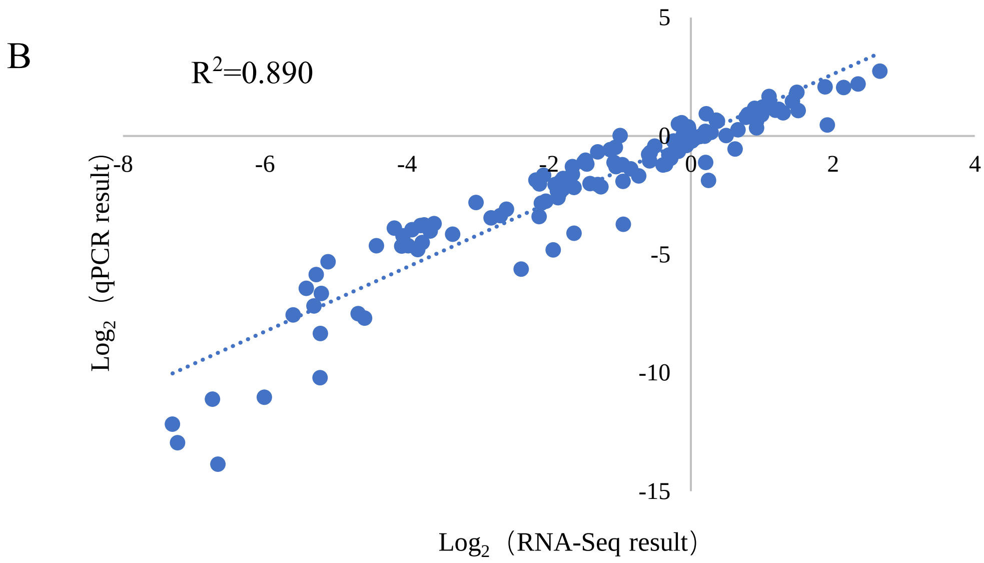

Fig. S4 The correlation between RNA-seq and qPCR results for naturally growing tea leaves (A) and *in vitro* study (B).
